# Best anthropometric discriminators of incident type 2 diabetes among white and black adults: A longitudinal ARIC study

**DOI:** 10.1371/journal.pone.0168282

**Published:** 2017-01-31

**Authors:** Dale S. Hardy, Devita T. Stallings, Jane T. Garvin, Hongyan Xu, Susan B. Racette

**Affiliations:** 1 Institute of Public and Preventive Health, Augusta University, Augusta, Georgia, United States of America; 2 School of Nursing, Saint Louis University, St. Louis, Missouri, United States of America; 3 College of Nursing, Augusta University, Augusta, Georgia, United States of America; 4 Department of Biostatistics and Epidemiology, Augusta University, Augusta, Georgia, United States of America; 5 Program in Physical Therapy and Department of Medicine, Washington University School of Medicine, St. Louis, Missouri, United States of America; McMaster University, CANADA

## Abstract

**Objective:**

To determine which anthropometric measures are the strongest discriminators of incident type 2 diabetes (T2DM) among White and Black males and females in a large U.S. cohort.

**Methods:**

We used Atherosclerosis Risk in Communities study data from 12,121 participants aged 45–64 years without diabetes at baseline who were followed for over 11 years. Anthropometric measures included a body shape index (ABSI), body adiposity index (BAI), body mass index (BMI), waist circumference (WC), waist to hip ratio (WHR), waist to height ratio (WHtR), and waist to hip to height ratio (WHHR). All anthropometric measures were repeated at each visit and converted to Z-scores. Hazard ratios and 95% confidence intervals adjusted for age were calculated using repeated measures Cox proportional hazard regression analysis. Akaike Information Criteria was used to select best-fit models. The magnitude of the hazard ratio effect sizes and the Harrell’s C-indexes were used to rank the highest associations and discriminators, respectively.

**Results:**

There were 1,359 incident diabetes cases. Higher values of all anthropometric measures increased the risk for development of T2DM (p < 0.0001) except ABSI, which was not significant in White and Black males. Statistically significant hazard ratios ranged from 1.26–1.63 for males and 1.15–1.88 for females. In general, the largest hazard ratios were those that corresponded to the highest Harrell’s C-Index and lowest Akaike Information Criteria values. Among White and Black males and females, BMI, WC, WHR, and WHtR were comparable in discriminating cases from non-cases of T2DM. ABSI, BAI, and WHHR were inferior discriminators of incident T2DM across all race-gender groups.

**Conclusions:**

BMI, the most commonly used anthropometric measure, and three anthropometric measures that included waist circumference (i.e., WC, WHR, WHtR) were the best anthropometric discriminators of incident T2DM across all race-gender groups in the ARIC cohort.

## Introduction

Obesity is one of the major risk factors for type 2 diabetes (T2DM). Approximately 85% of the U.S. population with T2DM is either overweight or obese [1]. If this trend continues, one in three adults in the U.S. will have diabetes by 2050 [1]. Due to growing rates of obesity and severe obesity, it is essential to understand the role of body fat distribution and the utility of anthropometric measures in discriminating incident cases from non-cases of T2DM. Anthropometric measures serve as proxies to visceral and subcutaneous abdominal adipose tissues, which are associated with insulin resistance and metabolic abnormalities [2,3]. However, these metabolic abnormalities may differ across race-gender groups. In the abdominal cavity, visceral adipose tissue compared to subcutaneous adipose tissue, contains a larger number of inflammatory and immune cells that are to linked to impaired glucose, abnormal lipid metabolism, and all-cause mortality [4,5]. In cross-sectional studies, waist circumference (WC) was shown to be a better predictor of visceral and subcutaneous adipose tissues among White and Black men and women than body mass index (BMI) [3,6]. However, at higher values of BMI and WC, visceral adipose tissue volume is greater in White men and women than in Black men and women [7]. Furthermore, subcutaneous adipose tissue volume tends to be higher in women than men [7].

Anthropometric measures are used frequently to examine the relationships between T2DM and obesity. However, it is controversial as to which measure best predicts future T2DM in specific race-gender groups [8–10]. The aim of this study was to compare seven anthropometric measures to determine the strongest discriminators of incident T2DM among White and Black males and females in a large U.S. cohort followed for more than 11 years.

## Methods

### Study sample

Participants were drawn from the Atherosclerosis Risk in Communities (ARIC) study, a large, ongoing, prospective cohort study designed to investigate the etiology of atherosclerosis and disease outcomes of adults residing in four U.S. communities: Baltimore, Maryland; Minneapolis, Minnesota; Jackson, Mississippi; and Winston-Salem, North Carolina. Approximately 4,000 individuals aged 45–64 years old were enrolled at each ARIC site between 1987 and 1989 [11,12]. All participants signed an informed consent document. Baseline de-identified ARIC data from 14,812 participants with and without T2DM were obtained from BioLINCC (https://biolincc.nhlbi.nih.gov/home/) [13]. The current secondary data analysis was approved by the Augusta University (formerly Medical College of Georgia) Institutional Review Board.

### Study variables

T2DM was the outcome variable, defined according to the American Diabetes Association criteria [14] by one or more the following: fasting blood glucose ≥ 126mg/dL, non-fasting blood glucose ≥ 200 mg/dL, self-reported diabetes diagnosis, or taking diabetes medications. Diabetes status and anthropometric data were collected with each study visit. The following seven anthropometric measures were included in our analysis: a body shape index [ABSI = waist circumference (cm) / (BMI^0.66^ x height (m)^0.5^)], body adiposity index [BAI = hip circumference (cm) / ((height (m)^1.5^) -18)], body mass index [BMI = body weight (kg) / height (m^2^)], waist circumference [WC, cm], waist to hip ratio [WHR = waist circumference (cm) / hip circumference (cm)], waist to height ratio [WHtR = waist circumference (cm) / height (cm)], and waist to hip to height ratio [WHHR = WC (cm) / (hip circumference (cm)/ height (cm))]. These variables were calculated from body weight, height, WC, and hip circumference measured in the fasted state, in light clothing without shoes, by trained ARIC technicians. WC was measured using an anthropometric tape at the level of the umbilicus with the participant standing [15]. Hip circumference was measured at the maximal protrusion of the buttocks. Race identification and gender were self-reported. Age (continuous) was the only variable considered for covariate adjustment. We tested the inclusion of variables for cigarette smoking (current, former, never (yes/no)) and alcohol intake (g/day) in the models. However, these variables did not appreciably decrease the Akaike Information Criteria (AIC) of their respective models, and therefore were excluded from the final analysis.

Physical activity using the sports domain of the Baecke questionnaire responses [16] collected at visits one and three only, and highest education level completed (<high school, high school graduate to some college or vocational school graduate, and ≥ college graduate), collected at visit one only were used to describe the sample population at baseline, but were not included in the final analysis because they were not collected at all four study visits. Other participant characteristics used to describe the sample population at baseline, but not included in the final analysis were fasting blood glucose, total cholesterol, HDL cholesterol, LDL cholesterol, triglycerides, and use of blood pressure medications (yes/no). Time to development of T2DM (cases) and survival time for non-cases were used in analyses.

### Statistical analysis

Participants were excluded from analysis if they had diabetes at baseline (n = 1808), if they were missing baseline data for anthropometric measures (n = 14), or did not return for any follow-up visits (n = 869). The remaining sample for analysis consisted of 12,121 participants. For each race-gender group, all anthropometric measures were converted to Z-scores using the following equation: Z-score = (individual anthropometric value—group mean)/ group SD anthropometric value. We used repeated measures Cox proportional hazard regression analysis to determine the risk of developing T2DM for each anthropometric measure. Survival time for development of T2DM was calculated with right-censoring as the mid-point of the time-interval from the visit when the participant was a non-case to the visit when they first met the criteria for diabetes. Time accumulated to the end of the study was calculated for participants who remained non-cases or were lost to follow-up in our sample. All seven anthropometric measures plus age, diabetes status, and survival time were available at all four study visits.

The AIC was used to assess the quality of the estimate with the step-wise addition of age in each anthropometric model. Anthropometric measures with the smallest AIC values were considered best-fit anthropometric measure models within race-gender groups. Harrell’s C-index, a rank parameter, was used as a measure of general predictive power of the Cox proportional regression model [17]. Harrell’s C-index was constructed by regressing T2DM on each anthropometric measure adjusted for age in separate anthropometric models, then assessing the fit of the model. Anthropometric models with the highest Harrell’s C-index concordance areas were chosen as the best-fit discriminators of anthropometric measures within race-gender groups. To determine the anthropometric measures with the highest associations, we ranked the hazard ratios within race-gender groups by their magnitude of effect from most to least, and contrasted this with their corresponding Harrell’s C-indexes.

We further tested the equality of Harrell’s C-index concordance areas of the pair-wise comparable discriminatory ability of the best-fit anthropometric measure with each of the other anthropometric measures (six pairs) within race-gender groups, by examining the Harrell’s C-index and its p value [17] with Bonferroni correction for multiple testing [18] Statistical analyses were conducted using Stata MP, Version 14.0 (StataCorp, College Station, Texas, U.S.). For most analyses, p <0.05 was considered statistically significant; for Bonferroni multiple comparisons testing [18], p < 0.008 (0.05/6) was used. All analyses for time-to development of T2DM, including hazard ratios, Harrell’s C-indexes, and AIC statistics were bootstrapped 5000 times with robust covariance structure and the efron method of ties.

## Results

Participants included 12,121 adults with a mean age of 54 (SD 5.7) years at baseline who did not have T2DM. Table 1 shows baseline characteristics by race-gender group. Black men and women were slightly younger, had higher HDL cholesterol levels, greater use of blood pressure medication, and lower levels of physical activity compared to White men and women. The majority of the sample was either overweight (40.3%) or obese (23.6%). Black men generally had lower anthropometric values than White men, despite equivalent BMI values. Black women had higher anthropometric values than White women. There were 1,359 (11.21%) incident T2DM cases over 11.85 years of follow-up: 522 (11.73%) White males, 157 (15.39%) Black males, 410 (8.13%) White females and 270 (16.77%) Black females. For those who had T2DM over the course of the study, the mean/maximum time to development of T2DM was 3.01/9.10 years for White males, 2.87/8.59 years for Black males, 3.02/8.10 years for White females, and 2.58/10.76 years for Black females. We tested for the presence of outliers for the anthropometric measures and their clinical characteristics. We decided to leave these outliers in our sample because they represented biologically plausible clinical criteria for diagnosis of T2DM.

**Table 1:** Baseline characteristics of participants without diabetes: The ARIC study.

	Range	Whites Males	Black Males	White Females	Black Females
		(n = 4451)	(n = 1020)	(n = 5040)	(n = 1610)
Characteristics		Mean (SD) or Column % of Sample			
Age (years)	44–66	54.6 (5.7)	53.5 (5.9)*	53.9 (5.7)	52.8 (5.7)§
Sports physical activity (Baecke units)	1–5	2.7 (0.8)	2.3 (0.7)*	2.4 (0.8)	2.1 (0.6)§
Fasting blood glucose (mg/dL)	50–125	101 (8.8)	99 (10.0)*	97 (8.7)	98 (10.1)§
Total cholesterol (mg/dL)	68–594	211 (37.9)	211 (43.4)	217 (40.9)	216 (45.1)
HDL cholesterol (mg/dL)	12–163	44 (12.3)	51 (16.8)*	59 (16.9)	60 (17.8)§
LDL cholesterol (mg/dL)	13–505	140 (35.3)	138 (41.5)	135 (39.2)	136 (43.6)
Triglycerides (mg/dL)	24–400	134 (66.6)	108 (55.5)*	118 (60.0)	98 (43.2)§
Blood pressure medications (%)	yes/no	21.9	32.9*	24.0	43.8§
Alcohol intake (g)	0–1856	72.0 (121.1)	72.5 (147.4)	25.1 (54.1)	11.4 (42.3)§
Cigarette smoking, current, former, never (%)	yes/no	23.9, 47.3, 28.8	37.9*, 33.8*, 28.2	24.4, 25.0, 50.6	24.9, 17.3§, 57.7§
Education level (%)					
< High school	yes/no	16.3	40.6*	14.7	36.0§
High school or vocational school graduate or some college education	yes/no	39.3	26.5*	51.4	30.1§
≥College graduate	yes/no	44.5	33.0*	33.9	33.9
Anthropometric Measures					
ABSI (cm/(kg/m^2^)^0.66^/cm^0.5^)	0.0414–0.1119	0.0845 (0.0032)	0.0816 (0.0038)*	0.0840 (0.0059)	0.0817 (0.0059)§
Body adiposity index (cm/m^1.5^–18)	7.7–67.2	25.8 (3.3)	25.8 (4.0)	32.3 (5.3)	34.8 (6.3)§
BMI (kg/m^2^)	14.4–65.9	27.2 (3.8)	27.4 (4.6)	26.2 (5.1)	30.2 (6.5)§
Underweight, normal weight, overweight (%)	yes/no	0.2, 28.2, 51.7	1.1*, 30.2*, 43.6*	1.3, 47.6. 30.6	0.9, 18.3§, 37.0§
Classes I, II, III obesity (%)	yes/no	16.5, 2.7, 0.7	19.9, 3.6, 1.6*	13.8, 4.8, 1.9	24.8§, 11.6, 7.3§
Waist circumference (cm)	52–178	99.1 (10.1)	96.0 (12.1)*	91.9 (13.9)	98.4 (16.0)§
Waist to height ratio (cm/cm)	0.31–1.09	0.56 (0.06)	0.54 (0.07)*	0.57 (0.09)	0.60 (0.10)§
Waist to hip ratio (cm/cm)	0.491–1.393	0.966 (0.051)	0.934 (0.055)*	0.885 (0.078)	0.894 (0.080)§
Waist to hip to height ratio (cm/cm/cm)	0.0029–0.0080	0.0055 (0.0004)	0.0053 (0.0004)*	0.0055 (0.0005)	0.0055 (0.0005)

Values reflect mean (SD) or percent (%) of sample. Abbreviations: ABSI, a body shape index; BMI, body mass index; HDL, high density lipoprotein cholesterol; LDL, low density lipoprotein cholesterol; SD, standard deviation. Physical activity was calculated using the Baecke questionnaire responses for sport activities [16]. Sports physical activity, fasting blood glucose, total cholesterol, HDL cholesterol, blood pressure medications, alcohol intake, cigarette smoking, and educational level were calculated using a smaller sample size (White males (n = 4333), Black males (n = 968), White females (n = 4979), and Black females (n = 1533). Statistical comparisons between race-gender groups were made using Pearson’s chi-square tests for categorical variables and t-tests for continuous variables:

*p<0.0001 comparing Black males to White males;

^§^p<0.0001 comparing Black females to White females, except for HDL where p = 0.0117.

S1 Table shows the correlational relationships between the anthropometric measures. As expected, all measures of waist circumference (WC, WHtR, WHR, and WHHR) were strongly correlated with each other. Surprisingly, ABSI had strong correlations with WHHR among White and Black females, which were not present among males. BAI was strongly correlated with BMI, WC, and WHtR among males and females (except with WC for white males). BMI was strongly correlated with WC and WHtR but had mostly moderate correlations with WHR and WHHR among the race-gender groups.

**Fig 1:**
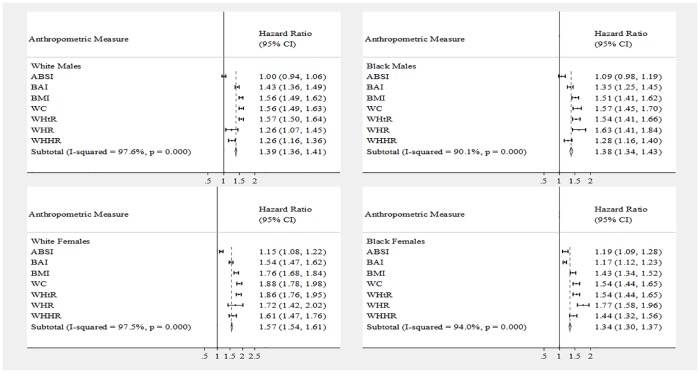
Forest plot of hazard ratios by race-gender groups: The ARIC study. Abbreviations: ARIC, Atherosclerosis Risk in Communities; a body shape index (ABSI), body adiposity index (BAI), body mass index (BMI), waist circumference (WC), waist to height ratio (WHtR), waist to hip ratio (WHR), and waist to hip to height ratio (WHHR). Individual models using repeated measures survival analysis were constructed with T2DM status (yes/no) as the response, with each anthropometric measure as the exposure variable, adjusted for age (5-year increments), over 4 visits (baseline: 1987–1989, visit 2: 1990–1992, visit 3: 1993–1995, visit 4: 1996–1998) using Atherosclerosis Risk in Communities study data.

Table 2 shows the hazard ratios for each anthropometric measure for incident T2DM, adjusted for age, for each race-gender group. All anthropometric measures were positively associated with risk of developing diabetes (p < 0.0001). For every one-unit increase in each anthropometric measure Z-score, the corresponding hazard ratio displays the increased risk for development of T2DM. Fig 1 presents a graphical view of the hazard ratios in a forest plot. In general, the largest hazard ratios were those that corresponded to the best-fit Harrell’s C-Index, shown in Table 3. Harrell’s C- indexes were largest for White females and smallest for Black females. Statistically significant hazard ratios ranged from 1.26–1.63 for males and 1.15–1.88 for females. The proportional hazards assumption was not violated in any model.

**Table 2:** Hazard ratios for incident type 2 diabetes, by anthropometric measure: The ARIC study.

		Hazard Ratio (95% Confidence Interval)		
Anthropometric Measure	Whites Males	Black Males	White Females	Black Females
	(n = 4451)	(n = 1020)	(n = 5040)	(n = 1610)
ABSI	1.00 (0.94–1.06)	1.09 (0.99–1.19)	**1.15 (1.08–1.22)**	**1.19 (1.09–1.29)**
Body adiposity index	**1.43 (1.36–1.49)**	**1.35 (1.25–1.45)**	**1.54 (1.47–1.62)**	**1.17 (1.12–1.23)**
BMI	**1.56 (1.49–1.63)**	**1.51 (1.41–1.63)**	**1.76 (1.68–1.84)**	**1.43 (1.35–1.52)**
Waist circumference	**1.56 (1.50–1.63)**	**1.57 (1.45–1.70)**	1.88 (1.78–1.98)*	**1.54 (1.44–1.65)**
Waist to height ratio	1.57 (1.50–1.64)*	**1.54 (1.42–1.66)**	**1.86 (1.76–1.96)**	**1.54 (1.44–1.65)**
Waist to hip ratio	**1.26 (1.08–1.47)**	1.63 (1.43–1.86)*	**1.72 (1.45–2.05)**	1.77 (1.59–1.97)*
Waist to hip to height ratio	**1.26 (1.17–1.36)**	**1.28 (1.17–1.40)**	**1.61 (1.47–1.77)**	**1.44 (1.33–1.57)**

Abbreviations: ARIC, Atherosclerosis Risk in Communities; ABSI, a body shape index; BMI, body mass index. **Bold** indicates hazard ratios that were statistically significant at **p < 0.0001**, except waist to height ratio among White males (p = 0.003). Individual models using repeated measures survival analysis were constructed with diabetes status (yes/no) as the response, with each anthropometric measure as the exposure variable, adjusted for age (5-year increments), over 4 visits (baseline: 1987–1989, visit 2: 1990–1992, visit 3: 1993–1995, visit 4: 1996–1998) using Atherosclerosis Risk in Communities study data.

* indicates highest hazard ratio (highest effect estimate).

**Table 3:** Harrell’s C-Index for incident type 2 diabetes by anthropometric measure: The ARIC study.

		Harrell’s C-Index (95% Confidence Interval)		
Anthropometric Measure	Whites Males	Black Males	White Females	Black Females
	(n = 4451)	(n = 1020)	(n = 5040)	(n = 1610)
ABSI	0.519 (0.496–0.523)	0.527 (0.497–0.556)	0.559 (0.537–0.582)	0.528 (0.506–0.550)
Body adiposity index	0.632 (0.616–0.649)	0.620 (0.589–0.650)	0.651 (0.633–0.670)	0.585 (0.562–0.608)
BMI	0.666 (0.650–0.682)*	0.666 (0.637–0.696)*	0.708 (0.690–0.726)*	**0.620 (0.598–0.642)**
Waist circumference	**0.654 (0.637–0.671)**	**0.665 (0.635–0.696)**	**0.701 (0.684–0.719)**	**0.628 (0.606–0.651**)
Waist to height ratio	**0.660 (0.644–0.676)**	**0.659 (0.629–0.689)**	**0.699 (0.681–0.717)**	**0.626 (0.604–0.649)**
Waist to hip ratio	**0.640 (0.624–0.657)**	**0.647 (0.618–0.676)**	0.708 (0.690–0.725)*	0.650 (0.628–0.672)*
Waist to hip to height ratio	0.597 (0.579–0.614)	0.567 (0.537–0.597)	0.645 (0.627–0.664)	0.589 (0.566–0.611)

Abbreviations: ARIC, Atherosclerosis Risk in Communities; ABSI, a body shape index; BMI, body mass index. **Bolded values** are not statistically different from best-fit Harrell’s C-Index* within each race-gender group using Bonferroni multiple testing criteria of p >0.008. Bonferroni p values were calculated as p = 0.05/6 (pairs) = 0.008. Individual models using repeated measures survival analysis were constructed with diabetes status (yes/no) as the response, with each anthropometric measure as the exposure variable, adjusted for age (5-year increments), over 4 visits (baseline: 1987–1989, visit 2: 1990–1992, visit 3: 1993–1995, visit 4: 1996–1998) using Atherosclerosis Risk in Communities study data.

*Best-fit Harrell’s C-index model (highest value).

We tested for differences in hazard ratios effect sizes of the anthropometric measures between race and gender separately using tests for interactions. BAI (p<0.0001), BMI (p = 0.044), WHtR (p = 0.009), and WHHR (p = 0.023) interacted with gender. BMI, WC, WHR, and WHtR interacted with race (all p values <0.0001). The results from these interactions suggest that there are differences between these anthropometric measures for females vs. males and Blacks vs. Whites for development of T2DM.

We observed similarities and differences between the hazard ratios for development of T2DM and Harrell’s C-indexes for discriminating T2DM cases from non-cases. Among White males, WHtR was the highest hazard ratio and Harrell’s C-index followed by BMI, WC and WHtR. Among Black males, WHR had the highest hazard ratio, but Harrell’s C-index was highest for BMI followed by WC and WHtR. The hazard ratio for WHR was lowest among these four measures for Black males. Therefore, among Black males, WHR was best at predicting T2DM, but poor at discriminating cases from non-cases for development of T2DM. Among White females, hazard ratios were highest for WC and similar for WHtR, followed by BMI and WHR. However, among White females, Harrell’s C-index ability to detect T2DM cases from non-cases was highest for BMI and WHR (over-lapping estimates), followed by WC and WHtR. Among Black females, WHR had the highest hazard ratio and Harrell’s C-index, followed by WC and WHtR (which had identical hazard ratios), and then BMI. For the Black female, WHR had the highest hazard ratio and was the best discriminator of T2DM. Based on the magnitude of the effect estimates of the hazard ratios and Harrell’s C-indexes, in general, ABSI, BAI, and WHHR had the weakest associations and discriminatory ability for T2DM across all race-gender groups (Table 3). Their hazard ratios ranged from 1.00–1.43 for males and 1.15–1.61 for females and Harrell’s C-indexes ranged from 0.519 to 0.632 for males and 0.528 to 0.645 for females.

We tested the inclusion of added covariates, physical activity (Baecke sports domain) [16] after imputing the missing values from visits 2 and 4, and education level from visit 1 only. We did not find any significant changes in the anthropometric effect estimates with these terms in the models, so we decided to keep age (5-year increments) as the only covariate in the model.

BMI, WC, WHR, and WHtR were comparable discriminators of T2DM across all race-gender groups (Table 3). Although BMI was the discriminator with the highest value, for White males, Black males, and White females, WC, WHR, and WHtR were comparable to BMI among all race-gender groups. The results of the AIC in S2 Table show the anthropometric discriminators with the lowest AIC values (i.e., best-fit model) across race-gender groups. These results are consistent with the results of comparability among the Harrell’s C-indexes. The anthropometric measures and corresponding lowest AIC values by race and gender for best-fit model are as follows: White males: BMI = 19715.38, Black males: WC = 4714.42, White females: WC = 14965.42, and Black females: WHR = 9339.83.

We sought to optimize the discrimination of incident T2DM to detect cases from non-cases by combining the best-fit anthropometric measure with other comparable anthropometric measures within each-race-gender group. There was only 1% improvement in the discriminatory improvement for White males with the addition of WHR to best-discriminator BMI model. Likewise, improvement in discrimination was also marginal for White females (3%) with the addition of WHR to best-fit BMI model, and 2% and 1.9% with the addition of WC and WHtR to best-fit WHR model, respectively. Similarly, among Black females, the discrimination of WHR was improved only by 1.2% with the addition of BMI or WC or WHtR, to the model. There was no improvement in discrimination for Black males.

## Discussion

In this longitudinal cohort of Black and White adults in the ARIC study with over 11 years of follow-up, we found that higher values of all anthropometric measures significantly increase the risk for development of T2DM. However, some anthropometric measures had higher associations and were better than others in discriminating cases from non-cases for incident T2DM, with differences by race and gender. WHtR had the highest association for incident T2DM among White males and WC was the highest association among White females. WHR was the highest association for incident T2DM among Black males and females. BMI was the best discriminator of incident T2DM among White males, Black males, and White females, while WHR was the best discriminator of incident T2DM among Black females. Furthermore, BMI, WC, WHR, and WHtR were comparable anthropometric discriminators to the best anthropometric discriminators of incident T2DM across all race-gender groups. In general, ABSI, BAI, and WHHR had lower associations and were inferior discriminators of incident T2DM among all race-gender groups.

In other longitudinal studies, WC alone or expressed as a ratio as WHR or WHtR, had the highest associations and were stronger discriminators of incident T2DM. Measures of central adiposity had the highest associations and higher discriminatory power for T2DM in African Americans, whereas BMI, WC, and WHtR had higher associations and were better discriminators in Whites [19]. Other reports showed that WC had the highest hazard ratios in men and women, but WHtR was the strongest anthropometric discriminator of incident T2DM in men and WC and WHtR were strongest in women [9]. In a Chinese cohort followed for 15 years, WC had the strongest discriminatory ability to detect cases from non-cases of incident T2DM, followed by BMI and ABSI [20]. Likewise, in an Aboriginal population, WC was the strongest discriminator of incident T2DM and cardiovascular disease among participants followed for up to 20 years [21,22]. Meta-analyses of longitudinal studies conducted in Europe, Australasia, Asia and the Middle-East revealed that WHtR was superior in discriminating cases from non-cases of incident T2DM, hypertension, and dyslipidemia [23,24]. In this current longitudinal study, there was only marginal improvement in discrimination of T2DM by adding more than one comparable anthropometric measure with the highest ranked best-fit anthropometric discriminator, BMI for White males and White females, and WHR for Black females. Furthermore, we found no improvement in discrimination of incident T2DM for Black males.

Some cross-sectional studies found that anthropometric measures of abdominal adiposity (i.e. WC, WHR, WHtR) were the best discriminators of T2DM [20,24,25]. Other studies support the value of these measures in morbidly obese persons, but did not find BMI to be a comparable discriminator of T2DM [26]. A cross-sectional study by gender found that WC and WHtR were better discriminators in women and WHR was a better discriminator among men. In this study, WC and WHtR had the strongest associations for T2DM and WHtR showed the strongest discriminator for future T2DM in men and women [27]. Another cross-sectional study in Iran revealed that WC followed by WHR, then BMI had the highest associations for T2DM in men, but in women, WHR had the highest association followed by WC, then BMI. However, WHR, followed by WHtR were better discriminators of T2DM in men and women [28]. In this same study, BAI was not associated with T2DM.

In our previous cross-sectional study [29], WC, WHtR, and WHR were the best discriminators of T2DM among White females, whereas in the current longitudinal analysis, BMI was an additional comparable discriminator of incident T2DM. Among Black females, WHR was the best-fit anthropometric discriminator both in the cross-sectional and the longitudinal studies. Unlike among the other race-gender groups, BMI was the best-fit anthropometric discriminator in the current longitudinal study. Similar to our cross-sectional study, the current longitudinal study also found that ABSI and BAI were inferior at discriminating T2DM in our sample. WHHR was an inferior discriminator in this current longitudinal study as well.

Although WC most accurately represents visceral adipose tissue [6,30] WC also represents subcutaneous fat [6,31,32]. Cross-sectional studies have found that the proportion of the body that represents visceral adipose tissue increases with age. On the other hand, subcutaneous adipose tissue increases with the level of obesity [32]. Anthropometric measures that reflect overall adiposity (i.e., BMI) and central adiposity (i.e., WC, WHR, WHtR) were the strongest discriminators of incident T2DM among middle-aged White and Black males and females in this ARIC study.

Our study had strengths as well as limitations. The large sample size enabled us to examine discriminators of incident T2DM within race-gender groups and representation from four different communities in the U.S. enhanced generalizability. Another strength was the length of follow-up, which spanned more than 11 years. Our study results are consistent with an earlier longitudinal ARIC study that used fewer anthropometric markers and receiver operator characteristic curves [33]. Although there was lack of information on the concrete date of diagnosis of T2DM, we believe that the diagnosis of T2DM in our participants was sound because patients brought in their medications at each study visit and their medical records were verified for new cases of T2DM diagnosis. Another potential limitation is that the last data collection occurred 15 years ago. However, we do not believe that this has diminish the scientific value of our findings because we used the current criteria for diagnosis of T2DM [14], and the sample included individuals with classes I, II, and III obesity (BMI ≥30 kg/m^2^). Furthermore, at baseline, classes II and III obesity (BMI ≥35 kg/m^2^) were highest in Black females (18.9%; n = 305/1610). This suggests that our study may have broad application to this race-gender group for those meeting the criteria for higher classes of obesity. For the Black female, WHR in particular should be monitored closely in the clinical setting. We recommend that clinicians use BMI and other measures of central obesity. Taken together, our findings suggest that more complicated formulas including ABSI, BAI and WHHR offer no advantage over the traditional measure of BMI and the less complicated measures of WC, WHtR, and WHR. Clinicians should monitor BMI along with WC, WHtR, and WHR to assess all adults for signs associated with the risk of of future T2DM.

In summary, BMI and anthropometric measures of central obesity that included WC were the strongest anthropometric discriminators for incident T2DM among White and Black males and females in a large U.S. cohort.

## Supporting information

S1 Table Correlations between anthropometric measures by race and gender: The ARIC study. All correlations had p values at p < 0.0001, except where *, p value = 0.0017 and §, p value was not significant. Correlations were computed for Pearson linear correlational relationships for a body shape index (ABSI), body adiposity index (BAI), body mass index (BMI), waist circumference (WC), waist to height ratio (WHtR), waist to hip ratio (WHR), and waist to hip to height ratio (WHHR) within race-gender groups for Whites and African Americans in the Atherosclerosis Risk in Communities (ARIC) study. Strong and weak correlations are highlighted in dark grey and light grey, respectively. Correlations from 0 to < 0.3 = poor correlation; ≥ 0.3 to < 0.7 = moderate correlation; ≥ 0.7 to 1.0 = strong correlation.
(DOCX)

S2 Table Akaike Information Criteria for incident type 2 diabetes by anthropometric measure: The ARIC study. Abbreviations: ARIC, Atherosclerosis Risk in Communities; ABSI, a body shape index; BMI, body mass index. *Best-fit Akaike Information Criteria (lowest value). Individual models using repeated measures survival analysis were constructed with diabetes status (yes/no) as the response, with each anthropometric measure as the exposure variable, adjusted for age (5-year increments), over 4 visits (baseline: 1987–1989, visit 2: 1990–1992, visit 3: 1993–1995, visit 4: 1996–1998) using Atherosclerosis Risk in Communities study data.
(DOCX)
