# Supplementary material for: Best anthropometric discriminators of incident type 2 diabetes among white and black adults: A longitudinal ARIC study
Source: PLoS One. 2017 Jan 31;12(1):e0168282. doi: 10.1371/journal.pone.0168282 (PMC5283673; doi:10.1371/journal.pone.0168282)
Supplement: S1 Table — All correlations had p values at p < 0.0001, except where *, p value = 0.0017 and §, p value was not significant. Correlations were computed for Pearson linear correlational relationships for a body shape index (ABSI), body adiposity index (BAI), body mass index (BMI), waist circumference (WC), waist to height ratio (WHtR), waist to hip ratio (WHR), and waist to hip to height ratio (WHHR) within race-gender groups for Whites and African Americans in the Atherosclerosis Risk in Communities (ARIC) study. Strong and weak correlations are highlighted in dark grey and light grey, respectively. Correlations from 0 to < 0.3 = poor correlation; ≥ 0.3 to < 0.7 = moderate correlation; ≥ 0.7 to 1.0 = strong correlation. (DOCX) [file pone.0168282.s001.docx]

**S1 Table. Correlations between anthropometric measures by race and gender: The ARIC study.**

|  |  |  | **White Males** | | |  |  |
| --- | --- | --- | --- | --- | --- | --- | --- |
|  | **ABSI** | **BAI** | **BMI** | **WC** | **WHtR** | **WHR** | **WHHR** |
| **ABSI** | 1.00 |  |  |  |  |  |  |
| **BAI** | -0.07 | 1.00 |  |  |  |  |  |
| **BMI** | -0.18 | **0.82** | 1.00 |  |  |  |  |
| **WC** | 0.26 | 0.68 | **0.88** | 1.00 |  |  |  |
| **WHtR** | 0.22 | **0.85** | **0.90** | **0.93** | 1.00 |  |  |
| **WHR** | 0.20 | 0.30 | 0.53 | 0.60 | 0.62 | 1.00 |  |
| **WHHR** | 0.34 | 0.39 | 0.37 | 0.39 | 0.62 | 0.63 | 1.00 |
|  |  |  | **Black Males** | | |  |  |
|  | **ABSI** | **BAI** | **BMI** | **WC** | **WHtR** | **WHR** | **WHHR** |
| **ABSI** | 1.00 |  |  |  |  |  |  |
| **BAI** | -0.05* | 1.00 |  |  |  |  |  |
| **BMI** | -0.14 | **0.86** | 1.00 |  |  |  |  |
| **WC** | 0.29 | **0.74** | **0.89** | 1.00 |  |  |  |
| **WHtR** | 0.28 | **0.86** | **0.90** | **0.95** | 1.00 |  |  |
| **WHR** | 0.23 | 0.36 | 0.54 | 0.61 | 0.62 | 1.00 |  |
| **WHHR** | 0.52 | 0.35 | 0.32 | 0.45 | 0.62 | 0.56 | 1.00 |
|  |  |  | **White Females** | | |  |  |
|  | **ABSI** | **BAI** | **BMI** | **WC** | **WHtR** | **WHR** | **WHHR** |
| **ABSI** | 1.00 |  |  |  |  |  |  |
| **BAI** | -0.01§ | 1.00 |  |  |  |  |  |
| **BMI** | -0.04 | **0.88** | 1.00 |  |  |  |  |
| **WC** | 0.48 | **0.72** | **0.84** | 1.00 |  |  |  |
| **WHtR** | 0.47 | **0.80** | **0.85** | **0.97** | 1.00 |  |  |
| **WHR** | 0.31 | 0.35 | 0.47 | 0.57 | 0.59 | 1.00 |  |
| **WHHR** | **0.73** | 0.28 | 0.33 | 0.62 | **0.71** | 0.56 | 1.00 |
|  |  |  |  | | |  |  |
|  |  |  | **Black Females** | | |  |  |
|  | **ABSI** | **BAI** | **BMI** | **WC** | **WHtR** | **WHR** | **WHHR** |
| **ABSI** | 1.00 |  |  |  |  |  |  |
| **BAI** | 0.00§ | 1.00 |  |  |  |  |  |
| **BMI** | -0.05 | **0.89** | 1.00 |  |  |  |  |
| **WC** | 0.45 | **0.74** | **0.85** | 1.00 |  |  |  |
| **WHtR** | 0.44 | **0.82** | **0.86** | **0.97** | 1.00 |  |  |
| **WHR** | 0.40 | 0.26 | 0.41 | 0.57 | 0.57 | 1.00 |  |
| **WHHR** | **0.72** | 0.25 | 0.29 | 0.58 | 0.66 | 0.61 | 1.00 |
