# Supplementary material for: Best anthropometric discriminators of incident type 2 diabetes among white and black adults: A longitudinal ARIC study
Source: PLoS One. 2017 Jan 31;12(1):e0168282. doi: 10.1371/journal.pone.0168282 (PMC5283673; doi:10.1371/journal.pone.0168282)
Supplement: S2 Table — Abbreviations: ARIC, Atherosclerosis Risk in Communities; ABSI, a body shape index; BMI, body mass index. *Best-fit Akaike Information Criteria (lowest value). Individual models using repeated measures survival analysis were constructed with diabetes status (yes/no) as the response, with each anthropometric measure as the exposure variable, adjusted for age (5-year increments), over 4 visits (baseline: 1987–1989, visit 2: 1990–1992, visit 3: 1993–1995, visit 4: 1996–1998) using Atherosclerosis Risk in Communities study data. (DOCX) [file pone.0168282.s002.docx]

**S2 Table. Akaike Information Criteria for incident type 2 diabetes by anthropometric measure: The ARIC study.**

|  |  | **Akaike Information Criteria** | | |  | |
| --- | --- | --- | --- | --- | --- | --- |
| **Anthropometric Measure** | **Whites Males** | | **Black Males** | **White Females** | | **Black Females** |
|  | (n = 4451) | | (n = 1020) | (n = 5040) | | (n = 1610) |
| ABSI | 20052.62 | | 4810.52 | 15366.79 | | 9489.70 |
| Body adiposity index | 19817.77 | | 4765.28 | 15153.42 | | 9445.44 |
| BMI | **19715.38*** | | 4727.23 | 14972.58 | | 9407.27 |
| Waist circumference | 19739.30 | | **4714.42*** | **14965.42*** | | 9380.23 |
| Waist to height ratio | 19716.76 | | 4726.01 | 14972.34 | | 9379.24 |
| Waist to hip ratio | 19897.92 | | 4725.33 | 15039.78 | | **9339.83*** |
| Waist to hip to height ratio | 19955.61 | | 4792.63 | 15182.46 | | 9440.04 |
